# Mechanisms of potentiation of Angiotensin II-induced contractile response of isolated rat aorta by hydrogen peroxide and tert-butyryl hydroperoxide

**DOI:** 10.4103/0253-7613.55208

**Published:** 2009-06

**Authors:** R. J. Patel, P. D. Patel, M. M. Patel, N. J. Patel, B. Thyagarajan

**Affiliations:** Department of Pharmacology, Shree S. K. Patel College of Pharmaceutical Education and Research, Ganpat Vidyanagar, Kherva-382711, Gujarat, India; 1 Department of Pharmacology and Physiology, UMDNJ - New Jersey Medical School, Medical Sciences Building, 185 S, Orange Avenue, Newark, NJ 07103

**Keywords:** Angiotensin II, hydrogen peroxide, reactive oxygen species, tert butyryl hydroperoxide

## Abstract

**Objective:**

To study the mechanism involved in hydrogen peroxide (H_2_O_2_) or tert-butyl hydroperoxide (t-BHP)-induced potentiation of the Ang II-mediated contraction of isolated rat thoracic aorta.

**Materials and Methods:**

Thoracic aorta was isolated from the *Sprauge dawley* rats (300–320 gm), cut spirally and response to Ang II (5 × 10^−8^M) was taken in the absence and presence of H_2_O_2_ (10^−6^M) and t-BHP (10^−5^M). To explore the probable mechanism of H_2_O_2_ and t-BHP-induced potentiation of Ang II-mediated contractile response, different blockers such as losartan (AT_1_ receptor blocker; 1 μM), catalase (H_2_O_2_ scavenger; 500 U/ml), lercanidipine (L-type calcium channel blocker; 1 μM), geinistein (tyrosine kinase inhibitor; 100 μM), and indomethacin (cyclo-oxygenase inhibitor; 10 μM) were used.

**Results:**

In spiral preparation of rat thoracic aorta, H_2_O_2_ (10^−6^M) and t-BHP (10^−5^M) did not produce the contraction as such. However, when they are added simultaneously with Ang II (5 × 10^−8^ M), they potentiated the contractile response of the Ang II. Catalase (500 U/ml) partially antagonized the Ang-II-induced contraction, as well as antagonized the potentiation induced by H_2_O_2_. Losartan (1 μM) and lercanidipine (1 μM) antagonized the Ang II-induced contractile response without affecting H_2_O_2_ (10^−6^M)-mediated potentiation. Geinistein (100 μM) antagonized H_2_O_2_ (10^−6^M)-mediated potentiation, but it slightly decreased the Ang II response. Losartan (1 μM) and lercanidipine (1 μM) and Geinistein (100 μM) antagonized the Ang II-induced contractile response but not t-BHP-mediated potentiation. Indomethacin antagonized t-BHP-mediated potentiation without affecting much of Ang II response.

**Conclusion:**

From the above-mentioned results, we can reasonably conclude that H_2_O_2_ and t-BHP potentiated the contraction induced by the Ang II. H_2_O_2_-induced potentiation of Ang II response may be mediated through tyrosine kinase activation and t-BHP through the activation of cyclo-oxygenase enzyme.

## Introduction

Several intracellular signaling events stimulated by reactive oxygen species (ROS) have been defined, including the activation of mitogen-activated protein kinase family, tyrosine kinases, and different isoenzymes of protein kinase C (PKC) as redox-sensitive kinases.[1–3] Plethora of literature supported that ROS may directly induce cell proliferation of some cell types.[4–6]

Ang II directly causes cell proliferation and cell hypertrophy of different cell types,[7] stimulates gene transcription,[8] induces gene expression of enzymes that produce mediators (e.g. NAD(P)H oxidase, phospholipase A and phospholipase D)[9,10] and also activates multiple intracellular signaling cascades (Tyrosine kinases, Ca^2+^-calmodulin, and mitogen-activated protein (MAP) kinases). Moreover, the hypertrophic effect of the peptide on vascular cells may be prevented by antioxidant treatments. These actions of Ang II have been related to increased ROS synthesis, which is dependent on peptides.[11] Specific studies directed to assess the specific pathway of ROS synthesis activated by Ang II point to H_2_O_2_ as the main ROS involved in the hypertrophic response and to the NADH/NADPH oxidase system as the primary target for peptide action.[12] There is only one study, as far as we know, which reported that H_2_O_2_ may play a role in Ang II-mediated contraction *in vitro*; however, the concentration response relation and underlying mechanism, especially for the potentiation component were not fully documented.[13]

In light of the above observations, the present experiments were designed to study the role of ROS, particularly hydrogen peroxide and t-butyryl hydroperoxide in Angiotensin II-induced contractile response in isolated rat thoracic aorta.

## Materials and Methods

### Chemicals

Angiotensin II and catalase were purchased from MP Biomedicals Inc, France; t-BHP and geinistein from Sigma Chemicals Co., St. Louis, USA; H_2_O_2_ from Merc, India. All other chemicals were of reagent grade, and were received as gift sample.

### Animals

Healthy and adult male *Sprague Dawley* rats (300–320 g) were procured from Central Animal Facility, SKPCER. They were maintained under controlled temperature and humidity, and standard diet and water was provided *ad libitum*. The care and the use of these animals were in accordance with the guidelines of the CPCSEA. Institutional Animal Ethics Committee approved the experimental protocols.

### Rat thoracic aorta spiral preparation

Rats were sacrificed by cervical dislocation under mild anesthesia and thoracic aorta was isolated from the heart to the diaphragm. It was freed from fat and connective tissues. Care was taken not to stretch the vessel. Helical strips of aorta of 3 mm in width and 20 mm in length were cut with sharp iris scissors and placed in 10-ml organ bath containing a modified Krebs Henseleit solution (KHS; NaCl – 118 mM, KCl – 4.7 mM, KH_2_PO_4_ – 1.2 mM, MgSO_4_.7H_2_O – 1.2 mM, CaCl_2_.2H_2_O – 2.5mM, NaHCO_3_ – 25 mM and glucose – 11 mM) of pH 7.4 (at every hour pH was checked and adjusted) and osmolality (280-308 mmol/kg). The solution was continuously aerated with carbogen (95% O_2_ + 5% CO_2_) at 37°C. A resting tension of 2 g was applied and allowed to equilibrate for 2 h. Changes in the isotonic contraction were recorded on chart recorder using an isotonic fine-movement transducer (Bio Devices, Ambala, India). During equilibration and throughout the experiment, KHS in the organ bath was changed in every fifteen min.

Protocol for vascular reactivity study

After 2 h of equilibration period, two wake up responses of KCl (80 mM) were taken to check the stability of tissue. Responses of Ang II (5 × 10^−8^M) were taken in the absence and presence of H_2_O_2_ (10^−6^M) and t-BHP (10^−5^M). For deciphering mechanism behind the potentiation of Ang II by H_2_O_2_ and t-BHP, they were added when the Ang II responses had reached the platue in the absence and presence of blocker. The preparation was preincubated with the blocker for 15 min.

### Statistical analysis

The responses were expressed as mean th ± SEM of % contraction and *n* represents the number of rats. Statistical analysis was performed by ANOVA. The differences were considered as significant when *P*<0.05.

## Results

The direct addition of H_2_O_2_ (10^−6^M) and t-BHP (10^−5^M) to isolated rat thoracic aorta did not produce contraction excluding their direct contractile effect.

**Figure 1:**
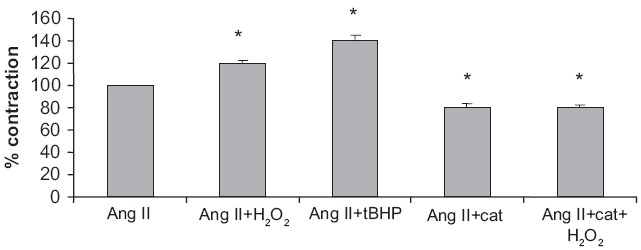
Potentiation of Ang II by H_2_O_2_ and t-BHP and effect of catalase on H_2_O_2_-induced potentiation. Ang II (5 × 10^−8^ M), H2O2 (10^−6^M), t-BHP (10^−5^ M) and catalase (500 U/ml). Values are expressed as mean ± SEM from six animals. *P<0.05 compared with Ang II alone

In the present experiments, the contraction by Ang II (5 × 10^−8^M)-induced aorta was used as a control. We studied the contraction of Ang II (5 × 10^−8^M) preincubated aorta with H_2_O_2_ (10^−6^M) and t-BHP (10^−5^M). The contraction of aorta elicited by Ang II was augmented after ROS treatment. Catalase (500 U/ml) partially arrested the Ang II-mediated contraction. There was also suppression of the potentiation of Ang II by H_2_O_2_ after catalase treatment [Figure 1].

**Figure 2:**
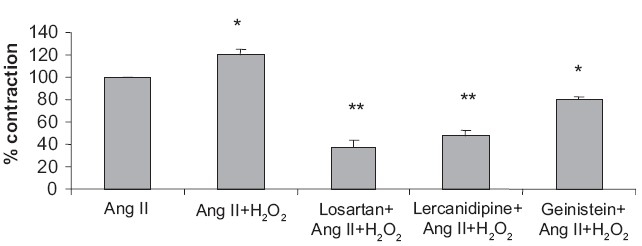
Effect of losartan, lercanidipine, and geinistein on H_2_O_2_-induced potentiation of contractile response to Ang II in rat aorta. Ang II (5 × 10^−8^ M), H_2_O_2_ (10^−6^ M), Losartan (1 μM), lercanidipine (1 μM), and Geinistein (100 μM). Values are expressed as mean + SEM from six animals.*P<0.05, **P<0.01 compared with Ang II alone

To explore the possible mechanism of H_2_O_2_-induced potentiation of Ang II contractile response, the effect of various blockers to its responses were studied in the absence and the presence of H_2_O_2._ Losartan (1 μM) and lercanidipine (1 μM) antagonized the Ang II-induced contractile response, but not antagonized H_2_O_2_-induced potentiation of Ang II contractile response. Geinistein (100 μM) significantly attenuated H_2_O_2_ -induced potentiation of Ang II contractile response and also partially antagonized Ang II-induced contractile response [Figure 2].

**Figure 3:**
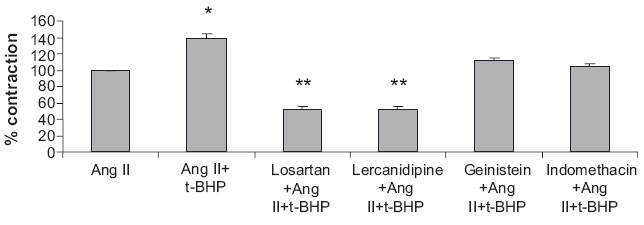
Effect of losartan, lercanidipine, geinistein, and indomethacin on t-BHP-induced potentiation of contractile response to Ang II in rat aorta. Ang II (5 × 10^−8^ M), t-BHP (10^−5^ M), losartan (1 μM), lercanidipine (1 μM), geinistein (100 μM), and indomethacin (10 μM). Values are expressed as mean ± SEM from six animals. *P<0.05, **P<0.01 compared with Ang II alone.

Similarly, losartan (1 μM) and lercanidipine (1 μM) blocked the Ang II-induced contractile responses, but not t-BHP-induced potentiation of Ang II-induced contractile response. Geinistein (100 μM) failed to suppress t-BHP-induced potentiation of Ang II contractile responses, but it partially antagonized Ang II-induced contractile response. Indomethacin (10 μM) attenuated the t-BHP-induced potentiation without affecting much of Ang II responses [Figure 3].

## Discussion

In the present study, we found that H_2_O_2_ and t-BHP potentiated the contractile response of Ang II. This study was designed to explore the probable mechanisms of this potentiation.

The current experimental findings suggest that H_2_O_2_ may have a role in the contraction of Ang II in rat thoracic aorta, because catalase completely blocked the H_2_O_2_-induced potentiation of contractile response to Ang II and also suppressed the contractile response induced by Ang II. The best-recognized action of catalase is its ability to remove hydrogen peroxide,[14] and as catalase blocked the Ang II -induced contraction, it can be hypothesized that H_2_O_2_ generation is necessary for the Ang II-induced contractile response. The argument, which supports this hypothesis is that the synthesis of H_2_O_2_ probably increased after Ang II treatment in aorta may be responsible as exogenously administered H_2_O_2_ (10^−6^M), did not induce any contractile response in quiescent aorta, while it potentiated the contractile response of Ang II in precontracted aorta. Previous studies strongly support the idea that the NADH/NADPH oxidase system may be the target for an increased production of hydrogen peroxide after Ang II treatment.[12] The results obtained in the present study may also involve the activation of NADP/NADPH oxidase pathway for Ang II-induced contractile response.

Losartan and lercanidipine produced partial antagonism of the Ang II-induced contractile response, suggesting that the Ang II-induced contraction is dependent not only on the classical pathways described previously but also it may be mediated through intracellular signal transduction, such as the activation of extracellular signal-regulated kinases.[15] Ang II-induced synthesis of hydrogen peroxide could play a central role, because it is well known that this ROS may also activate extracellular signal-regulated kinases.[16]

Losartan and lercanidipine failed to attenuate the H_2_O_2_-induced potentiation of Ang II, suggesting that AT_1_ receptor and Ca^2+^ channel activation may not be involved in H_2_O_2_-induced potentiation. In quiescent vessel, H_2_O_2_ at higher concentration induced vasoconstriction, which may be mediated through activation of phospholipase A_2_, cyclo-oxygenase, phospholipase C, and tyrosine kinase.[17,18] In the present study, the transient contractile component of Ang II-precontracted thoracic aorta to H_2_O_2,_ was significantly attenuated by a tyrosine kinase inhibitor, geinistein, suggesting the involvement of tyrosine kinase pathway as mediator in the potentiation activity.

Similarly, losartan and lercanidipine partially antagonized Ang II-induced contractile responses without affecting t-BHP-induced potentiation of Ang II-induced contractile response of rat aorta strip, suggesting that there is no involvement of AT_1_ receptor and Ca^2+^ channel in t-BHP-induced potentiation. Geinistein also did not modify the t-BHP-induced potentiation of Ang II contractile response. Indomethacin markedly suppressed t-BHP-induced potentiation without antagonism of Ang II-induced contractile response, which supports the work by Garcia-Cohen E.C. *et al*, 2000.[19] In short, it is demonstrated that H_2_O_2_ and t-BHP-induced potentiation of Ang II-induced contractile responses of isolated rat thoracic aorta may involve activation of tyrosine kinase and cyclo-oxygenase pathways, respectively.
